# Data on euglyphid testate amoeba densities, corresponding protozoic silicon pools, and selected soil parameters of initial and forested biogeosystems

**DOI:** 10.1016/j.dib.2018.10.164

**Published:** 2018-11-10

**Authors:** Daniel Puppe, Manfred Wanner, Michael Sommer

**Affiliations:** a Leibniz Centre for Agricultural Landscape Research (ZALF), Eberswalder Str. 84, 15374 Müncheberg, Germany; b Brandenburg University of Technology Cottbus-Senftenberg, Department Ecology, 03013 Cottbus, Germany; c University of Potsdam, Institute of Earth and Environmental Sciences, 14476 Potsdam, Germany

**Keywords:** Silicon cycling, Biogenic silica, Terrestrial biogeosystems, Biosilicification, Euglyphida

## Abstract

The dataset in the present article provides information on protozoic silicon (Si) pools represented by euglyphid testate amoebae (TA) in soils of initial and forested biogeosystems. Protozoic Si pools were calculated from densities of euglyphid TA shells and corresponding Si contents. The article also includes data on potential annual biosilicification rates of euglyphid TA at the examined sites. Furthermore, data on selected soil parameters (e.g., readily-available Si, soil pH) and site characteristics (e.g., soil groups, climate data) can be found. The data might be interesting for researchers focusing on biological processes in Si cycling in general and euglyphid TA and corresponding protozoic Si pools in particular.

**Specifications table**

**Table:** 

Subject area	*Biology*
More specific subject area	*Biogeochemistry*
Type of data	*Tables*
How data was acquired	*Soil analyses (for details see*Section 2.2.*) and microscopical examinations (for details see*Section 2.3.*)*
Data format	*Analyzed data*
Experimental factors	*Soil parameters, euglyphid testate amoeba densities, and corresponding protozoic silicon pools*
Experimental features	*Analyses of initial (chronosequence) and forest sites*
Data source location	*Germany, for latitudes & longitudes and further site details see*Table 1.
Data accessibility	*All data are presented within the paper.*

**Value of the data**

• The provided dataset is useful for comparison with the results of other authors regarding, e.g., ecological (euglyphid testate amoeba densities) and biogeochemical (protozoic silicon pools, annual biosilicification) issues in different global ecosystems.
• Data on protozoic silicon pools and corresponding annual biosilicification rates might emphasize the need for detailed investigations of silicon (re-)cycling in unicellular organisms in general and testate amoeba in particular (e.g., qualitative characterization of biogenic silicon, isotope analysis).
• Together with other datasets the presented data allow meta-analyses to examine significant controls (steady state soils) and dynamics (initial soils) of euglyphid testate amoeba densities and related amoebal biosilicification processes in soils in detail.
• In combination with data of other authors the presented data can be used for modelling to assess the role of euglyphid testate amoebae (compared to other organisms that synthesize biogenic silicon, e.g., plants or diatoms) for silicon cycling in soils and corresponding silicon fluxes from terrestrial to aquatic ecosystems.

## 1 Data

Diverse unicellular and multicellular organisms are able to synthesize structures of amorphous silica (SiO_2_·*n*H_2_O) [1]. In soils these structures represent different biogenic silicon (BSi) pools depending on their origin [2], [3]. In general, BSi plays an important role in the global cycling of Si [4], [5], [6]. However, while research has been focused on plantal Si and corresponding phytogenic Si pools since decades [e.g., [7], [8], [9]], far less is known about other BSi pools, e.g., protozoic Si pools represented by euglyphid testate amoeba (TA) shells [3], [10], [11], [12], [13], [14], [15]. Euglyphid TA represent a monophyletic clade (Euglyphida) of unicellular soil protists with a self‐secreted siliceous test (shell) and a worldwide distribution [16].

The presented data were the basis for analyses of protozoic Si pools in initial [12] and forested biogeosystems [3]. The dataset in the present article provides information on i) site characteristics, geographic positions, and climate data of the initial and forested biogeosystems (Table 1), ii) analyzed soil parameters (Table S1), iii) densities of euglyphid TA shells in soils (Table S2), and iv) corresponding protozoic Si pools as well as annual biosilicification rates of euglyphid TA at these sites (Table S3).

**Table 1:** Site characteristics, geographic positions, and climate data of the examined initial and forested biogeosystems. Data on precipitation and temperature represent annual averages for the period 1981–2010 (German Meteorological Service).

**Site**	**Lithology**	**Soil group (WRB)**	**Humus form**	**Ecosystem**	**Coverage (%)**			**Geographic position**		**Altitude**	**Precipitation**	**Temperature**
					**Tree**	**Shrub**	**Herb**	**Latitude**	**Longitude**	**(m a.s.l.)**	**(mm)**	**(°C)**
**CC**	Spoil of Quaternary sands	Arenosol	–	Initial (artificial catchment)	–	–	–	51°36′18′′ N	14°15′58′′ E	135	568	9.6
**NL**	Spoil of Quaternary sands	Arenosol	–	Initial (artificial catchment)	–	–	–	51°35′50′′ N	14°17′22′′ E	132	568	9.6
**AB**	Calcareous glacial till	Stagnosol	Mull	Forest (beech, oak)	68	3	70	54°12′23′′ N	13°02′21′′ E	34	654	8.6
**EG**	Dolomitic limestone	Cambisol	Mull	Forest (beech)	65	0	78	48°20′19′′ N	09°25′42′′ E	786	808	9.5
**HE**	Basalt	Vertisol	Mull	Forest (beech, ash, spruce, maple)	65	3	60	47°47′53′′ N	08°45′08′′ E	703	820	9.2
**MR**	Calcareous loess	Luvisol	Mull	Forest (beech)	85	35	50	49°07′13′′ N	08°40′38′′ E	217	776	10.0
**PP**	Calcareous loess	Stagnosol	Mull	Forest (pine, beech)	70	0	2	48°51′17′′ N	08°46′14′′ E	473	889	9.5
**ZE**	Kaolinitic claystone	Stagnosol	Mull	Forest (hornbeam, oak)	85	10	60	48°38′37′′ N	09°33′13′′ E	390	1090	9.9
**HS**	Siliceous sandstone	Podzol	Moder	Forest (pine, spruce)	73	18	63	48°34′21′′ N	08°20′60′′ E	832	1607	8.1
**RO**	Calcareous glacial till	Luvisol	Moder	Forest (beech, oak)	70	5	5	53°30′38′′ N	13°21′43′′ E	71	530	8.7
**SL**	Eolian sands (dune)	Arenosol	Moder	Forest (pine, beech)	80	0	5	49°20′51′′ N	08°38′14′′ E	123	861	11.0
**HK**	Siliceous sandstone	Planosol	Peat	Forest (spruce, pine)	65	15	60	48°34′13′′ N	08°20′53′′ E	907	1607	8.1

## 2 Experimental design, materials and methods

### 2.1 Site description and sampling scheme

#### 2.1.1 Initial sites

**Fig. 1:**
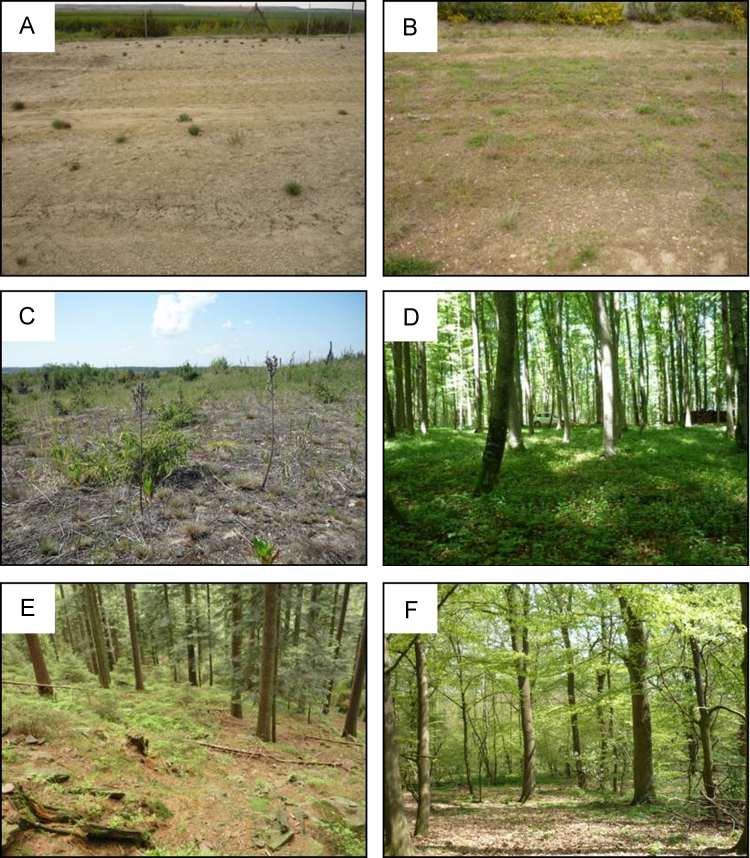
Photographs of initial (a-c) and some selected forest sites (d-f). A) CC (3-year-old), b) CC (5-year-old), c) NL (10-year-old), d) EG, e) HS, and f) RO. For site details see Table 1 and Section 2.1.

The artificial catchments ‘Chicken Creek’ (CC) and ‘Neuer Lugteich’ (NL) are part of a post-mining landscape located in the active mining area ‘Welzow-South’ (lignite open-cast mining, 150 km south-east of Berlin) in the state of Brandenburg, Germany. Climate is characterized by an average air temperature of 9.6 °C with an annual precipitation of 568 mm comprising data from 1981 to 2010. The construction of CC was completed in 2005 (time zero). In 2008 a small area in the west of the catchment was again restored to time zero (removal of the upper 20 cm of soil) for additional experimental plots. Construction of NL was finished in 2001 (time zero). Soils classify as Protic Arenosol (Calcaric, Transportic) or Haplic Arenosol (Hyperochric, Transportic) depending on site age [17]. Detailed information on site construction of CC and NL can be found in Gerwin et al. [18] and Kendzia et al. [19], respectively. All samples were taken from Quaternary substrate at 3-, 5- (CC), and 10-year-old (NL) spots representing a chronosequence (Fig. 1a-c). Samples (20 cm × 20 cm × 5 cm; subdivided in two compartments: 0–2.5 and 2.5–5 cm depth) were taken at randomly chosen spots within an area of approx. 25 m^2^. Vegetated (cov) and uncovered (unc) spots were sampled in four field replicates each to analyze possible impacts of vegetation (3 cov: *Tussilago farfara* and *Trifolium arvense*; 5 cov: *Corynephorus canescens* and *T. arvense*) on protozoic Si pools. At NL almost the whole surface was vegetated with biogenic crusts, Poales, and several shrubs, which is why only vegetated spots (10 cov) were sampled. Samples were taken in May 2010 (CC: 5 unc, 5 cov), May 2011 (NL: 10 cov), and August 2011 (CC: 3 unc, 3 cov).

#### 2.1.2 Forest sites

Ten non-eroded forest sites showing huge differences in climate, parent material, and pedogenesis were selected. Mean annual precipitation rates range from 530 to 1600 mm, mean annual air temperatures from 8 to 11 °C. Soils comprise (i) a sandy Arenosol developed from eolian sands (SL, dune), a Podzol and a Planosol from siliceous sandstones (HS, HK) very low in weatherable minerals (<10% feldspars, mica), (ii) silty to loamy Luvisols and Stagnosols from calcareous, illitic loess (MR, PP) and sandy to loamy Luvisols and Stagnosols from glacial till (RO, AB), both parent materials with intermediate contents of weatherable minerals (feldspars, mica), (iii) a clayey Cambisol from dolomitic limestone (EG), (iv) a clayey Stagnosol from kaolinitic claystone (ZE), and finally (v) a clayey, smectitic Vertisol from basalt (HE) very high in weatherable minerals, like augite and plagioclase. The forest stands are old and are assumed to be in steady state in terms of TA dynamics at a decadal time scale (photographs of some selected forest sites can be found in Fig. 1d-f). Soil samples were taken in four field replicates (*n* = 4) at all sample sites except HK (*n* = 3). The field replicates (20 cm × 20 cm each) were placed randomly within an area of approx. 100 m^2^. Per field replicate samples were taken in the upper 5 cm (incl. organic layers except for fresh litter) differentiating between two superimposed soil compartments about the same size (ideally 20 cm × 20 cm × 2.5 cm each) and transferred to plastic bags. Sampling took place in spring 2010 within six weeks (April 26th–June 6th).

### 2.2 Soil parameters

Bulk densities (BD, g cm^−3^) were calculated by dividing weights of oven-dried (105 °C) aliquots of soil samples by corresponding volumes. Remaining soil samples were air dried and sieved (2 mm) to separate fine-earth (<2 mm) from skeleton content (>2 mm). For soil analyses only fine-earth was used.

#### 2.2.1 Soil pH, carbon, and nitrogen

Soil pH was measured using a glass electrode in a 0.01 M CaCl_2_ solution with a soil-to-solution ratio of one-to-five. For total carbon as well as nitrogen analyses (C_t_ and N_t_) fine-earth samples were finely powdered in a disc mill. Subsequently, C_t_ and N_t_ were determined by dry combustion using an elemental analyzer (CNS TruSpec, Leco Instruments). Total inorganic carbon (TIC) was measured with a multiphase analyzer (RC 612, Leco Instruments). Soil organic carbon (SOC) concentrations were calculated by subtraction (C_t_-TIC) and C:N ratios were calculated by division (SOC:N_t_). Soil C and N analyses were performed at the minimum of two lab repetitions per sample.

#### 2.2.2 Readily-available silicon

For extraction of the calcium chloride (CaCl_2_) soluble, so-called readily- or plant-available Si fraction (Si_CaCl2_), 2 *g* of soil was mixed with 20 ml of a 0.01 MCaCl_2_ solution per sample and continuously shaken for 16 h using a lab roller mixer [20]. This Si fraction was extracted to characterize the Si supply for shell synthesis of euglyphid TA in soils. Subsequent to extraction, the extracts were centrifuged (4000 rpm, 30 min), filtered using 0.45 μm polyamide membrane filters, and Si concentrations were determined by ICP-OES (iCAP 6300 Duo, Thermo Scientific). Complete extraction work was done using plastic equipment only and results represent arithmetic means of three lab repetitions per sample.

#### 2.2.3 Data conversion and calculation steps

All results except for pH were converted to an oven-dry basis (105 °C). Fine-earth mass (FEM in kg m^−2^) was calculated considering bulk density, thickness and skeleton content (wt%). Total FEM (FEM_t_) of the upper 5 cm was calculated as the sum of FEM of superimposed compartments. For the upper 5 cm of soil pH was averaged as follows: Per compartment pH was multiplied with the corresponding FEM, divided by FEM_t_ and subsequently these results were summed up. Mass densities (g m^−2^) of SOC, N_t_, and Si_CaCl2_ were calculated compartment-wise by multiplying FEM with element concentrations (g kg^−1^). Finally, the results of superimposed compartments were summed up for the upper 5 cm of soil.

### 2.3 Euglyphid testate amoeba densities, protozoic silicon pools, and annual biosilicification

Soil samples in the plastic bags were homogenized by gentle manual mixing and subsequently 2 g of fresh soil was taken per sample for TA analyses and stored in 8 ml of formalin (4%). Soil suspensions received from serial dilution (1000–31.25 mg soil in 8 ml of water each) were stained with aniline blue. TA were enumerated using an inverted microscope (OPTIKA XDS-2, magnifications of 200× and 400×) differentiating between full (living incl. encysted individuals, stained) and empty shells (unstained) of the order Euglyphida. TA densities (shells cm^−2^) were calculated considering TA shell numbers (g^−1^ dry weight), bulk density (g cm^−3^), and thickness (cm) per soil compartment. TA densities of the upper 5 cm were calculated by summing up the corresponding TA densities of superimposed soil compartments.

For calculation of protozoic Si pools we differentiated between different TA taxa of the order Euglyphida with known silica contents per shell as published by Aoki et al. [10]. Summarizing these data we calculated Si contents (pg shell^−1^, in parentheses listed below) by simple multiplication (SiO_2_ content × 28/60 = Si content) for 9 TA taxa: *Assulina muscorum* (750), *Corythion dubium* (580), *Euglypha* spec. (720), *Euglypha rotunda/laevis* type (420), *Euglypha strigosa* type (1420), *Tracheleuglypha dentata* (750), *Trinema complanatum* (500), *Trinema enchelys* (770), and *Trinema lineare* (360). Indistinctly euglyphid shells or other silica platelet synthesizing TA taxa (e.g., *Valkanovia elegans*) were recorded as ‘euglyphid TA’ (700 pg Si per shell, mean of the Si content per shell of the 9 taxa above).

Protozoic Si pools (BSi_TA_; mg m^−2^) were calculated per soil compartment using the following formula:

(1) $${\mathrm{BSi}}_{\mathrm{TA}}=\sum_{i=1}^{n}{(N}_{i}\;\times \;{\mathrm{Si}}_{i}\;\times \;\rho_{b}\;\times \;t\;\times \;10^{-5})$$

where *N*_*i*_ is the number of euglyphid TA shells (g^−1^ dry weight), Si_*i*_ is the corresponding Si content (pg shell^−1^; given in parentheses listed above), *ρ*_*b*_ is the bulk density (g cm^−3^), and *t* is the thickness (cm) of the corresponding soil compartment. In contrast, for estimation of annual biosilicification only living euglyphid TA (g^−1^ dry weight) were considered for *N*_*i*_ in Eq. (1) due to their ability of reproduction. After calculation steps as described in Eq. (1) results were multiplied with 13 and 90 (potential TA generations per year, see Foissner [21]) for minimal and maximal annual biosilicification rates, respectively. For calculation of protozoic Si pools and euglyphid TA biosilicification rates of the upper 5 cm of soil the results of superimposed soil compartments were added up.

## Appendix A Supplementary material

**Table S1** Data on analyzed soil parameters of the upper 5 cm of soils (U = upper, L = lower soil compartment) at the initial and forested sites. BD = bulk density, SC = skeleton content, FEM = fine-earth mass, Si_CaCl2_ = readily-available silicon, SOC = soil organic carbon, N_t_ = total nitrogen.

.

**Table S2** Densities of euglyphid TA shells (E = empty, F = full shells) in the upper 5 cm of soils (U = upper, L = lower soil compartment) of the examined initial and forested biogeosystems.

.

**Table S3** Data on protozoic Si pools (T = total protozoic Si pools, E = protozoic Si pools represented by empty shells only, F = protozoic Si pools represented by full shells only) and potential minimal (Min) and maximal (Max) annual biosilicification rates of euglyphid TA.
